# Frontotemporal networks and behavioral symptoms in primary progressive aphasia

**DOI:** 10.1212/WNL.0000000000002579

**Published:** 2016-04-12

**Authors:** Lucio D'Anna, Marsel M. Mesulam, Michel Thiebaut de Schotten, Flavio Dell'Acqua, Declan Murphy, Christina Wieneke, Adam Martersteck, Derin Cobia, Emily Rogalski, Marco Catani

**Affiliations:** From Natbrainlab, Department of Forensic and Neurodevelopmental Sciences (L.D., M.T.d.S., F.D., M.C.), Department of Neuroimaging (F.D.), and Sackler Institute of Translational Neurodevelopment (D.M.), Institute of Psychiatry, Psychology and Neuroscience (IoPPN), King's College London, UK; Neurology Clinic, Department of Experimental and Clinical Medical Sciences (L.D.), University of Udine Medical School; Department of Neurosciences (L.D.), “S. Maria della Misericordia” University Hospital, Udine, Italy; Cognitive Neurology and Alzheimer's Disease Center (M.M.M., C.W., A.M., D.C., E.R.) and Department of Neurology (M.M.M., A.M., D.C.), Northwestern University Feinberg School of Medicine, Chicago, IL; and Brain Connectivity and Behaviour, Brain and Spine Institute (M.T.d.S.), CNRS UMR 7225 INSERM-UPMC UMRS 1127 La Salpêtrière, Paris, France.

## Abstract

**Objective:**

To determine if behavioral symptoms in patients with primary progressive aphasia (PPA) were associated with degeneration of a ventral frontotemporal network.

**Methods:**

We used diffusion tensor imaging tractography to quantify abnormalities of the uncinate fasciculus that connects the anterior temporal lobe and the ventrolateral frontal cortex. Two additional ventral tracts were studied: the inferior fronto-occipital fasciculus and the inferior longitudinal fasciculus. We also measured cortical thickness of anterior temporal and orbitofrontal regions interconnected by these tracts. Thirty-three patients with PPA and 26 healthy controls were recruited.

**Results:**

In keeping with the PPA diagnosis, behavioral symptoms were distinctly less prominent than the language deficits. Although all 3 tracts had structural pathology as determined by tractography, significant correlations with scores on the Frontal Behavioral Inventory were found only for the uncinate fasciculus. Cortical atrophy of the orbitofrontal and anterior temporal lobe cortex was also correlated with these scores.

**Conclusions:**

Our findings indicate that damage to a frontotemporal network mediated by the uncinate fasciculus may underlie the emergence of behavioral symptoms in patients with PPA.

Patients with primary progressive aphasia (PPA) show a gradual decline in language functioning with a relative sparing of other cognitive domains.^1^ Although the aphasia is the major cause of impaired function, additional symptoms such as distress, sadness, apathy, and depression can be seen in almost half of patients with PPA, followed by changes in eating, aberrant motor behavior, agitation, disinhibition, and irritability.^1,2^ In keeping with the progressive neurodegenerative etiologies of both PPA and behavioral variant frontotemporal dementia (bvFTD), it is not surprising that the symptom overlap between these 2 syndromes becomes increasingly more prominent as the disease progresses. In fact, approximately 75% of patients with PPA eventually develop severe behavioral problems, whereas 65% of patients with bvFTD manifest clear language impairment.^3^

Among the 3 variants of PPA, patients with the semantic form, in which the anatomical hallmark is represented by a marked atrophy of the anterior temporal lobes,^3^ are at higher risk of developing behavioral symptoms compared with the other variants. Rohrer and Warren^4^ found that in addition to anterior temporal lobe atrophy, the most significant anatomical cortical changes in patients with PPA and behavioral symptoms occur in the orbitofrontal cortex.

The anterior temporal and orbitofrontal regions are directly linked by the uncinate fasciculus. Although the association between degeneration of the uncinate fasciculus and semantic deficits is well-documented,^5,6^ the role of the uncinate fasciculus in patients with PPA and behavioral symptoms is unknown.

The aim of our study was to determine, through a multimodal neuroimaging approach, the anatomical abnormalities underlying behavioral symptoms in patients with PPA. We used diffusion tensor imaging (DTI) tractography to assess the microstructural organization of the major association tracts connecting to the orbitofrontal cortex or the anterior temporal lobe.^7,8^ We also measured cortical thickness of the orbitofrontal cortex and the anterior temporal lobe to determine whether white matter degeneration correlates with the degree of cortical atrophy.

## METHODS

### Participants and clinical assessment.

Thirty-three patients with PPA and 26 healthy controls matched for age, sex, and handedness were enrolled through the Primary Progressive Aphasia Program at the Cognitive Neurology and Alzheimer's Disease Centre, Northwestern University Feinberg School of Medicine.

The diagnosis of PPA was based on at least a 2-year history of progressive, isolated deterioration of speech or language functions. All patients were then classified into 1 of the 5 PPA variants based on several recent diagnostic classification guidelines.^1,9,10^ Patients with PPA who presented a severe (e.g., Boston Naming Test <50%) single isolated language symptom (anomia or dyslexia) without fulfilling the criteria for the other variants were classified as unclassified variant.^10^ Patients with PPA with a combination of agrammatism and semantic impairments were classified as mixed PPA.^11–13^

The Frontal Behavioral Inventory (FBI), originally developed and standardized with the purpose of differentiating bvFTD from other dementias and quantifying the severity of behavioral symptoms,^14,15^ was used to assess behavioral symptoms.

The FBI is based on the evaluation of the patient's caregiver that for each item scores the severity of symptoms in a scale between 0 and 3 (0 = never, 1 = mild or occasional, 2 = moderate, 3 = severe or very frequent). The FBI is composed of 24 items divided into 12 items for negative behavior symptoms (FBI negative symptoms score) and 12 for positive symptoms (FBI positive symptoms score). The FBI negative symptoms score contains 3 items that evaluate behavioral symptoms in relation to language impairment (item 9, logopenia; item 10, aphasia and verbal apraxia; item 11, comprehension and semantic deficits). To effectively evaluate the behavioral symptoms in the patients with PPA, we subtracted these 3 language-related items from the FBI negative symptoms score and the FBI total scores. All statistical analyses were therefore performed using corrected scores for the total FBI and negative FBI.

### MRI acquisition, DTI, and data processing.

MRI acquisitions were carried out on a 3T Siemens Trio MRI system at the Centre for Translational Imaging, Northwestern University of Chicago. T1-weighted magnetization-prepared rapid gradient echo (MPRAGE) sequences were acquired with the following parameters: repetition time 2,300 ms; echo time 2.86 ms; flip angle, 9; field of view, 256 mm; 60 slices; slice thickness 1.0 mm, as previously described.^6^ FreeSurfer image analysis suite (version 4.5.0) (http://surfer.nmr.mgh.harvard.edu/) was used to measure cortical thickness on T1-weighted MPRAGE images. Measures of cortical thickness were obtained by estimating the closest distance between the gray/white matter boundary and the gray matter/CSF boundary at each vertex of the tessellated surface.^6,16^ Differences in cortical thickness between patients with PPA and healthy controls were shown on the entire surface area of the neocortex using an FDR of 0.001.^6,17^ Cortical thickness was measured from a 20-mm region of interest (ROI) placed in the most atrophic anterior temporal and lateral orbitofrontal cortex, within the cortical projections areas of the uncinate fasciculus.

For the tractography analysis, we acquired a total of 72 contiguous near-axial slices using an acquisition sequence fully optimized for diffusion imaging, providing isotropic (2 × 2 × 2 mm) resolution and whole head coverage. At each slice location, 8 images were acquired with no diffusion gradient applied, together with 60 diffusion-weighted images (*b* value of 1,000 s/mm^2^). Explore DTI (http://www.exploredti.com) was used to perform DTI processing and to correct simultaneously subject motion and geometrical distortions with reorientation of the *b* matrix. RESTORE function excluded the remaining outliers and robustly fitted the tensor model in all voxels of the brain.^18,19^ Fractional anisotropy and radial diffusivity maps were calculated and saved in nifti format. A spline interpolated streamline algorithm was used to perform whole brain tractography (stepsize 0.5 mm; fractional anisotropy threshold 0.15; angle threshold 35). Finally, the whole brain tractography was imported in TrackVis (http://www.trackvis.org) for visualization.^6^

### Tract-specific reconstructions and measurements.

TrackVis was used to perform the virtual in vivo dissection of the 3 tracts of interest according to previously published methods.^6,20–25^ Tractography dissections were obtained using manually defined ROIs on the orthogonal fractional anisotropy images. The following tracts were dissected as previously described.^7,21^

The uncinate fasciculus is a U-shaped bundle that arises in the temporal pole, lateral to the parahippocampal gyrus and amygdala. In the temporal lobe, the uncinate fasciculus is ventral to the inferior fronto-occipital fasciculus. Arching forward, the uncinate then enters the external capsule and splits into a ventrolateral and an anteromedial branch.^7^ The ventrolateral component ends in the anterior insula and lateral orbitofrontal cortex, while the anteromedial branch reaches the olfactory cortex, the medial orbitofrontal cortex, and the frontal pole.^7^ Dissections of the uncinate fasciculus were performed by placing a ROI in the anterior temporal lobe and a second ROI in the external/extreme capsule.^6,20,26^

The inferior fronto-occipital fasciculus originates from the inferior and medial surface of the occipital lobe. When the inferior fronto-occipital fasciculus leaves the temporal lobe, it reduces its section and its fibers come together when they reach the extreme/external capsule at higher level respect to the uncinate fasciculus. In the frontal lobe, the dorsolateral fibers of the inferior fronto-occipital fasciculus end mainly in the inferior frontal gyrus, while the most ventral fibers gather together, ending in the medial fronto-orbital region and frontal pole.^21,26^

Two ROIs were used to dissect the inferior fronto-occipital fasciculus, one placed in the occipital white matter and a second region in the external/extreme capsule.^20^

Finally, the inferior longitudinal fasciculus is a ventral associative bundle connecting the occipital and temporal lobes. To dissect the inferior longitudinal fasciculus, the first region was placed in the anterior temporal lobe and the second in the occipital white matter.^20,25^

For each tract of interest, number of streamlines, fractional anisotropy, mean diffusivity, and axial and perpendicular diffusivity were extracted as indices of microstructural composition and architecture of the brain tissue.^25^ The number of streamlines was considered a surrogate of tract volume and atrophy.^6^ In dementia syndromes, the number of streamlines is reduced according to the severity of the pathology and clinical symptoms.^6,27^

Fractional anisotropy is a quantitative index of the degree of anisotropy of the biological tissue and indirectly of microstructural integrity. Fractional anisotropy provides information about the biological properties and the microarchitecture of the white matter fibers. Reduced fractional anisotropy values have been reported in disorders characterized by demyelination, edema, or degeneration.^28^

Perpendicular and axial diffusivities correspond to the diffusivity along the principal directions of the diffusion tensor and are generally used to quantify changes due to axonal/myelin damage.^23,28^

Ten practice datasets were used to train the dissector (L.D.), who was blind to any information about the cortical thickness results and the identity of the participants. L.D. began the dissections for this study only when he achieved high reliability.

### Statistical analysis.

All statistical analyses were performed using SPSS (Chicago, IL) software (version 21). Independent-samples *t* tests were run to examine group differences in number of streamlines, fractional anisotropy, and axial and perpendicular diffusivity of the different tracts of interest. Bonferroni correction was applied to correct for multiple comparisons (threshold at *p* ≤ 0.001). All *p* values are provided uncorrected. We used one-way analysis of variance (ANOVA) between groups to compare the differences between PPA subtypes and controls. Rho Spearman analysis was used to describe the strength and direction of the linear relationship between severity of behavioral symptoms and tract-specific measurements.

### Standard protocol approvals, registrations, and patient consents.

For this study, we received approval from an ethical standards committee on human experimentation for any experiments using human participants. We obtained written informed consents from all patients (or guardians of patients) participating in this study.

## RESULTS

Demographic, clinical, and behavioral features of our sample are reported in tables 1 and 2. Among the patients with PPA, 8 received a descriptive diagnosis of logopenic variant, 8 of nonfluent/agrammatic variant, 7 of semantic variant (PPA-S), 2 of mixed variant, and 8 of unclassified/severe variant.^1,9,10^ The PPA-S group were younger compared to the other variants and had higher prevalence of behavioral symptoms as reported in the FBI total scores and FBI positive symptoms scores.

**Table 1:**
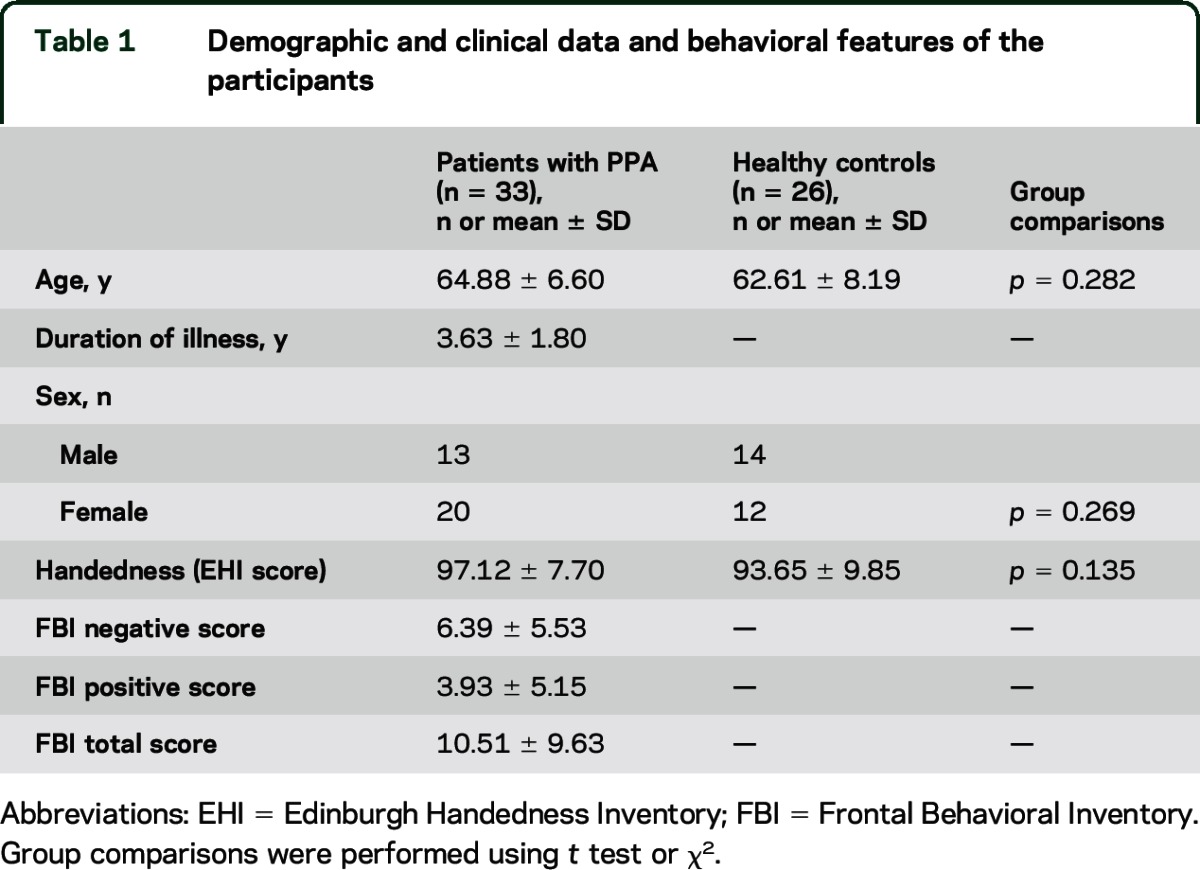
Demographic and clinical data and behavioral features of the participants

**Table 2:**
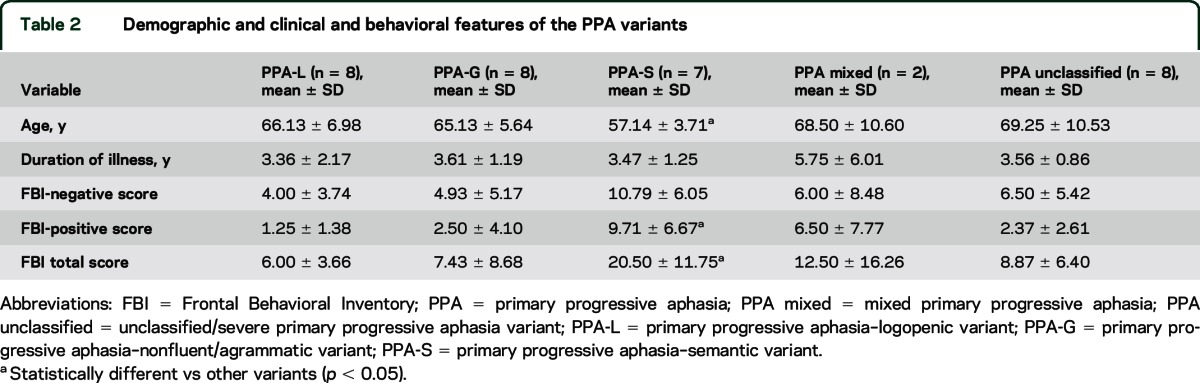
Demographic and clinical and behavioral features of the PPA variants

### White matter connections analysis.

**Figure 1:**
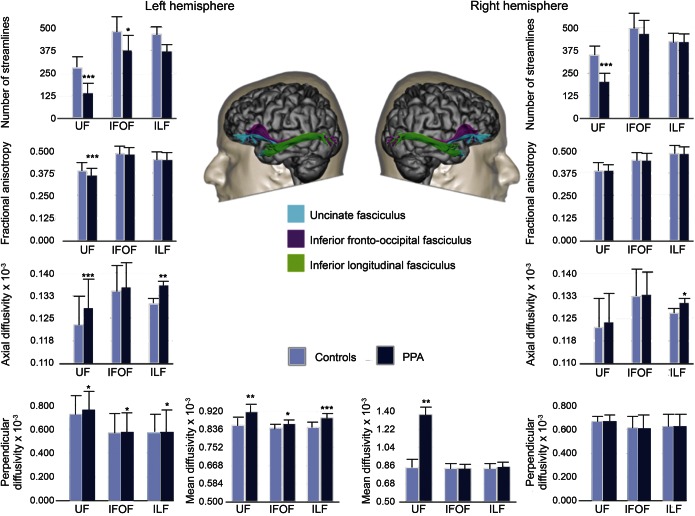
Tract-specific measurements Differences in tract-specific measurements of the uncinate fasciculus (UF), inferior fronto-occipital fasciculus (IFOF), and inferior longitudinal fasciculus (ILF) between controls and patients with primary progressive aphasia (PPA). Measurements of the number of streamlines, fractional anisotropy, mean diffusivity, axial diffusivity, and perpendicular diffusivity are reported for the tracts of interest. Statistically significant differences between controls and patients within each tract are indicated with asterisks (*p < 0.05; **p < 0.01; ***p < 0.001; Bonferroni threshold for significance = 0.0016).

After Bonferroni correction the left uncinate fasciculus of patients with PPA showed a significantly reduced number of streamlines (*p* < 0.001, *t*_[54]_ value = 7.942), lower fractional anisotropy (*p* < 0.001, *t*_[54]_ = 3.253), and a significant increase in axial (*p* < 0.001, *t*_[54]_ = −2.849) and perpendicular diffusivity (*p* = 0.020, *t*_[54]_ = −2.264) compared with healthy controls (figure 1, left). In the right hemisphere only the number of streamlines was significantly reduced also in the uncinate fasciculus of patients with PPA when compared with healthy controls (*p* < 0.001, *t*_[54]_ = 5.193) (figure 1).

ANOVA between PPA subtypes and controls showed statistically significant differences between groups in the number of streamlines (*F* = 4.933; *p* = 0.001), axial diffusivity (*F* = 5.038; *p* < 0.001), perpendicular diffusivity (*F* = 7.902; *p* < 0.001), and mean diffusivity (*F* = 7.243; *p* < 0.001). Abnormalities in the left uncinate fasciculus were particularly evident for the semantic subtype when compared with the other variants (figure e-1 on the *Neurology*® Web site at Neurology.org).

In the left uncinate fasciculus, the number of streamlines and fractional anisotropy were inversely correlated with total FBI scores (Spearman = −0.549, *p* = 0.001 and Spearman = −0.490, *p* < 0.001, respectively) and with both positive (Spearman = −0.530, *p* = 0.001 and Spearman = −0.500, *p* < 0.001, respectively) and negative FBI scores (Spearman = −0.460, *p* < 0.001 and Spearman = −0.450, *p* < 0.001, respectively), whereas axial and perpendicular diffusivity correlated directly with total FBI scores (Spearman = 0.450, *p* < 0.001 and Spearman = 0.540, *p* < 0.001, respectively) and with both positive (Spearman = 0.400, *p* < 0.001 and Spearman = 0.600, *p* < 0.001, respectively) and negative FBI scores (Spearman = 0.575, *p* < 0.001 and Spearman = 0.540, *p* < 0.001, respectively). These correlations indicate that behavioral symptoms are associated with poorer white matter integrity (table e-1, figure e-2). In the right uncinate fasciculus, axial diffusivity was correlated with negative (Spearman = 0.443, *p* = 0.005), positive (Spearman = 0.432, *p* = 0.005), and total FBI scores (Spearman = 0.421, *p* = 0.001); perpendicular diffusivity was correlated with FBI negative, positive, and total scores (Spearman = 0.497, *p* < 0.001; Spearman = 0.576, *p* < 0.001; Spearman = 0.580, *p* < 0.001) (table e-1). ANOVA between PPA subtypes and controls showed statistically significant differences in the number of streamlines (*F* = 4.840; *p* = 0.003) and axial diffusivity (*F* = 3.943; *p* < 0.04), which were particularly evident for the semantic subtype when compared to the other variants (figure e-1).

In the left inferior fronto-occipital fasciculus, the patients with PPA showed significantly fewer streamlines (*p* = 0.036, *t*_[54]_ = 2.256) and higher perpendicular diffusivity (*p* = 0.028, *t*_[54]_ = −2.145) when compared with healthy controls. No statistically significant differences were found for this tract in the right hemisphere (figure 1). ANOVA between PPA subtypes and controls did not show statistically significant differences between groups in terms of DTI measurements (figure e-3). We found no statistically significant correlations between any of the tractography measurements of the inferior fronto-occipital fasciculus and the scores of the FBI (table e-1).

In the inferior longitudinal fasciculus, patients with PPA showed a statistically significant increase of axial diffusivity in both sides (left: *p* = 0.002, *t*_[54]_ = −3.342; right: *p* = 0.030, *t*_[54]_ = −2.223) and perpendicular diffusivity in the left side (*p* = 0.020, *t*_[54]_ = −2.396) (figure 1). ANOVA between PPA subtypes and controls did not show statistically significant differences between groups in terms of DTI measurements (figure e-4). No significant correlations were found between diffusivity measurements of the inferior longitudinal fasciculus and scores on the FBI (table e-1).

### Cortical thickness analysis.

**Figure 2:**
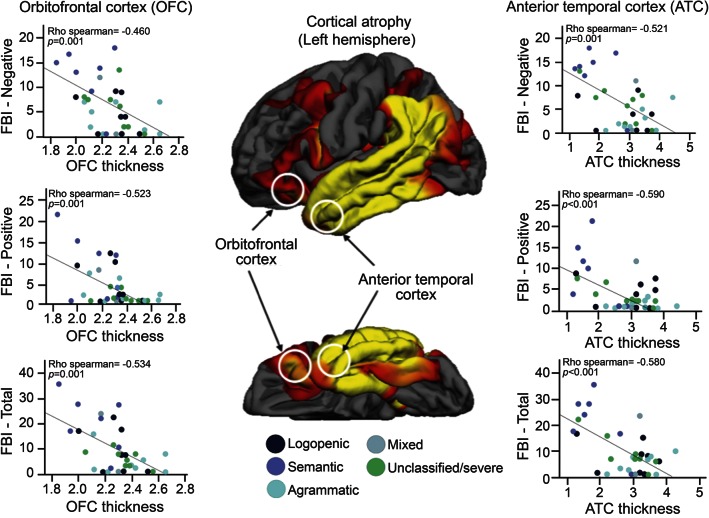
Correlations between left cortical thickness and Frontal Battery Inventory (FBI) scores The cortical thickness measurements of the left orbitofrontal cortex and anterior temporal lobe showed a statistically significant correlation with FBI negative, positive, and total scores.

A whole brain analysis showed significant cortical atrophy of the left temporal-parietal and frontal regions in the PPA compared with controls (figure 2). A ROI approach confirmed that compared with healthy controls, patients with PPA showed significant atrophy in the left (*p* < 0.001, *t*_[57]_ = 4.367) and right (*p* = 0.014, *t*_[57]_ = 2.537) orbitofrontal cortex and in the left (*p* = 0.001, *t*_[57]_ = 3.391) and right (*p* = 0.004, *t*_[57]_ = 2.996) anterior temporal lobe. Cortical thickness of the ROIs in the left orbitofrontal and anterior temporal cortex were inversely correlated with negative scores (Spearman = −0.460, *p* = 0.001 and Spearman = −0.521, *p* = 0.001, respectively), positive scores (Spearman = −0.523, *p* = 0.001 and Spearman = −0.590, *p* < 0.001, respectively), and total scores in the FBI (Spearman = −0.534, *p* = 0.001 and Spearman = −0.580, *p* < 0.001, respectively) (figure 2). Correlations between the right anterior temporal lobe and the scores on the FBI were less significant (table e-2).

In addition, cortical thickness measurements of both anterior temporal lobe and orbitofrontal cortex were directly correlated with fractional anisotropy (Spearman = 0.488, *p* = 0.003 and Spearman = 0.524, *p* = 0.001, respectively), and inversely correlated with axial diffusivity (Spearman = −0.570, *p* = 0.001 and Spearman = −0.378, *p* = 0.007, respectively) and perpendicular diffusivity (Spearman = −0.580, *p* = 0.001 and Spearman = −0.513, *p* = 0.002, respectively) (table e-3). Similar correlations were found for the right uncinate fasciculus (table e-4). No correlations were found between cortical thickness measurements and DTI measurements of the inferior fronto-occipital fasciculus and inferior longitudinal fasciculus (tables e-3 and e-4).

## DISCUSSION

Our findings showed that damage to a ventral frontotemporal network is correlated with behavioral symptoms in PPA (tables e-5 to e-8).

In previous studies,^29^ degeneration of the uncinate fasciculus has been found to correlate with behavioral symptoms in several other conditions affecting anterior temporal and orbitofrontal regions. In bvFTD, for example, the white matter damage in the uncinate fasciculus correlates with severity of apathy, impulsivity, inappropriate sexual behavior, and hoarding.^30^ In patients with idiopathic psychopathy,^22^ altered integrity of the uncinate fasciculus correlates with severity of antisocial behavior, which includes poor behavioral control, impulsivity, need for stimulation, proneness to boredom, lack of realistic goals, and irresponsibility. We had previously shown that damage to the uncinate fasciculus was correlated with semantic processing deficits in PPA.^5,6^ The current results reveal the additional association of this fasciculus with behavioral symptoms in this group of patients. Overall, these studies confirm that the uncinate fasciculus has a major role in a wide range of comportments and that its damage is associated with behavioral deficits irrespective of the presenting syndrome and underlying etiology.^7,31^

The uncinate fasciculus is the major association pathway between the anterior part of the temporal lobe, including the amygdala, and the ventral frontal (orbitofrontal) region.^20^ The temporopolar and ventral frontal cortices constitute multimodal convergence zones for sensory information.^32^ Here the stimuli are processed independently of their reward or punishment value.^33^ These cortical areas, and the amygdala with which they are interconnected, collectively play pivotal roles in multimodal integration as well as the motivational guidance and cognitive filtering of behavior.^30^ Our findings suggest that disruption of this network may underlie some of the behavioral abnormalities that emerge in PPA.^34,35^ The lack of a correlation between FBI scores and damage to the inferior fronto-occipital and inferior longitudinal fasciculi supports the relative specificity of this relationship. It appears therefore that the uncinate fasciculus has a dual functionality, one related to lexico-semantic processing^8^ and the other to a broad range of behaviors.^7^

In our study, we confirmed that among the patients with PPA, those with the semantic variant have the most severe damage to the uncinate fasciculus. Postmortem studies revealed that the most common pathology affecting the semantic variant is ubiquitin/TDP43-positive frontotemporal lobar degeneration, which is characterized by numerous dystrophic neurites associated with neuronal and synaptic loss.^10^ It is therefore most likely that diffusivity abnormalities in the uncinate fasciculus reflect axonal degeneration of the white matter fibers secondary to the cortical pathology.

Our findings confirm the existence of significant white matter damage in PPA^36^ and support the importance of anterior frontotemporal connections in sustaining normal behavior in humans. Future studies using higher resolution datasets and advanced methods for fiber crossing^37^ could help to isolate individual components of the uncinate fasciculus and their different functional correlates.

## Supplementary Material

Data Supplement

## GLOSSARY

ANOVA: analysis of variance
bvFTD: behavioral variant frontotemporal dementia
DTI: diffusion tensor imaging
FBI: Frontal Behavioral Inventory
MPRAGE: magnetization-prepared rapid gradient echo
PPA: primary progressive aphasia
PPA-S: primary progressive aphasia–semantic variant
ROI: region of interest

## References

[1] Mesulam MM Slowly progressive aphasia without generalized dementia. Ann Neurol 1982;11:592–598. 7114808 10.1002/ana.410110607

[2] Fatemi Y Boeve BF Duffy J Neuropsychiatric aspects of primary progressive aphasia. J Neuropsychiatry Clin Neurosci 2011;23:168–172. 21677245 10.1176/appi.neuropsych.23.2.168 PMC3204925

[3] Rosen HJ Allison SC Ogar JM Behavioral features in semantic dementia vs other forms of progressive aphasias. Neurology 2006;67:1752–1756. 17130406 10.1212/01.wnl.0000247630.29222.34

[4] Rohrer JD Warren JD Phenomenology and anatomy of abnormal behaviours in primary progressive aphasia. J Neurol Sci 2010;293:35–38. 20400120 10.1016/j.jns.2010.03.012 PMC2896484

[5] Galantucci S Tartaglia MC Wilson SM White matter damage in primary progressive aphasias: a diffusion tensor tractography study. Brain 2011;134:3011–3029. 21666264 10.1093/brain/awr099 PMC3187537

[6] Catani M Mesulam MM Jakobsen E A novel frontal pathway underlies verbal fluency in primary progressive aphasia. Brain 2013;136:2619–2628. 23820597 10.1093/brain/awt163 PMC3722349

[7] Catani M Dell'Acqua F Thiebaut de Schotten M A revised limbic system model for memory, emotion and behaviour. Neurosci Biobehav Rev 2013;37:1724–1737. 23850593 10.1016/j.neubiorev.2013.07.001

[8] Catani M Bambini V A model for Social Communication And Language Evolution and Development (SCALED). Curr Opin Neurobiol 2014;28:165–171. 25156623 10.1016/j.conb.2014.07.018

[9] Mesulam MM Primary progressive aphasia. Ann Neurol 2001;49:425–432. 11310619

[10] Gorno-Tempini ML Hillis AE Weintraub S Classification of primary progressive aphasia and its variants. Neurology 2011;76:1006–1014. 21325651 10.1212/WNL.0b013e31821103e6 PMC3059138

[11] Mesulam MM Weintraub S Is it time to revisit the classification guidelines for primary progressive aphasia? Neurology 2014;82:1108–1109. 24598706 10.1212/WNL.0000000000000272

[12] Mesulam MM Wieneke C Thompson C Rogalski E Weintraub S Quantitative classification of primary progressive aphasia at early and mild impairment stages. Brain 2012;135:1537–1553. 22525158 10.1093/brain/aws080 PMC3577099

[13] Mesulam M Wieneke C Rogalski E Cobia D Thompson C Weintraub S Quantitative template for subtyping primary progressive aphasia. Arch Neurol 2009;66:1545–1551. 20008661 10.1001/archneurol.2009.288 PMC2796598

[14] Kertesz A Nadkarni N Davidson W Thomas AW The frontal behavioral inventory in the differential diagnosis of frontotemporal dementia. J Int Neuropsychol Soc 2000;6:460–468. 10902415 10.1017/s1355617700644041

[15] Kertesz A Davidson W Fox H Frontal behavioral inventory: diagnostic criteria for frontal lobe dementia. Can J Neurol Sci 1997;24:29–36. 9043744 10.1017/s0317167100021053

[16] Fischl B Dale AM Measuring the thickness of the human cerebral cortex from magnetic resonance images. Proc Natl Acad Sci USA 2000;97:11050–11055. 10984517 10.1073/pnas.200033797 PMC27146

[17] Rogalski E Cobia D Harrison TM Wieneke C Weintraub S Mesulam MM Progression of language decline and cortical atrophy in subtypes of primary progressive aphasia. Neurology 2011;76:1804–1810. 21606451 10.1212/WNL.0b013e31821ccd3c PMC3100122

[18] Chang L-C Jones DK Pierpaoli C RESTORE: robust estimation of tensors by outlier rejection. Magn Reson Med 2005;53:1088–1095. 15844157 10.1002/mrm.20426

[19] Jones DK Basser PJ “Squashing peanuts and smashing pumpkins”: how noise distorts diffusion-weighted MR data. Magn Reson Med 2004;52:979–993. 15508154 10.1002/mrm.20283

[20] Catani M Thiebaut de Schotten M A diffusion tensor imaging tractography atlas for virtual in vivo dissections. Cortex 2008;44:1105–1132. 18619589 10.1016/j.cortex.2008.05.004

[21] Forkel SJ Thiebaut de Schotten M Kawadler JM Dell'Acqua F Danek A Catani M The anatomy of fronto-occipital connections from early blunt dissections to contemporary tractography. Cortex 2014;56:73–84. 23137651 10.1016/j.cortex.2012.09.005

[22] Craig MC Catani M Deeley Q Altered connections on the road to psychopathy. Mol Psychiatry 2009;14:1–8. 19506560 10.1038/mp.2009.40

[23] Catani M Thiebaut de Schotten M Atlas of Human Brain Connections. New York: Oxford University Press; 2012.

[24] Thiebaut de Schotten M Dell'Acqua F Valabregue R Catani M Monkey to human comparative anatomy of the frontal lobe association tracts. Cortex 2012;48:82–96. 22088488 10.1016/j.cortex.2011.10.001

[25] Catani M Occipito-temporal connections in the human brain. Brain 2003;126:2093–2107. 12821517 10.1093/brain/awg203

[26] Catani M Howard RJ Pajevic S Jones DK Virtual in vivo interactive dissection of white matter fasciculi in the human brain. Neuroimage 2002;17:77–94. 12482069 10.1006/nimg.2002.1136

[27] Catani M Diffusion tensor magnetic resonance imaging tractography in cognitive disorders. Curr Opin Neurol 2006;19:599–606. 17102700 10.1097/01.wco.0000247610.44106.3f

[28] Dell'Acqua F Catani M Structural human brain networks: hot topics in diffusion tractography. Curr Opin Neurol 2012;25:375–383. 22766720 10.1097/WCO.0b013e328355d544

[29] Oishi K Faria AV Hsu J Tippett D Mori S Hillis AE Critical role of the right uncinate fasciculus in emotional empathy. Ann Neurol 2015;77:68–74. 25377694 10.1002/ana.24300 PMC4293256

[30] Heide Von Der RJ Skipper LM Klobusicky E Olson IR Dissecting the uncinate fasciculus: disorders, controversies and a hypothesis. Brain 2013;136:1692–1707. 23649697 10.1093/brain/awt094 PMC3673595

[31] Ameis SH Catani M Altered white matter connectivity as a neural substrate for social impairment in Autism Spectrum Disorder. Cortex 2015;62:158–181. 25433958 10.1016/j.cortex.2014.10.014

[32] Mesulam M-M Principles of Behavioural and Cognitive Neurology. New York: Oxford University Press; 2000.

[33] Rolls ET Limbic systems for emotion and for memory, but no single limbic system. Cortex 2015;62:119–157. 24439664 10.1016/j.cortex.2013.12.005

[34] Hughes LE Rowe JB The impact of neurodegeneration on network connectivity: a study of change detection in frontotemporal dementia. J Cogn Neurosci 2013;25:802–813. 23469882 10.1162/jocn_a_00356 PMC3708294

[35] Tan RH Wong S Kril JJ Beyond the temporal pole: limbic memory circuit in the semantic variant of primary progressive aphasia. Brain 2014;137:2065–2076. 24844729 10.1093/brain/awu118

[36] Catani M Piccirilli M Cherubini A Axonal injury within language network in primary progressive aphasia. Ann Neurol 2003;53:242–247. 12557292 10.1002/ana.10445

[37] Dell'Acqua F Scifo P Rizzo G A modified damped Richardson–Lucy algorithm to reduce isotropic background effects in spherical deconvolution. Neuroimage 2010;49:1446–1458. 19781650 10.1016/j.neuroimage.2009.09.033

