# Supplementary material for: Frontotemporal networks and behavioral symptoms in primary progressive aphasia
Source: Neurology. 2016 Apr 12;86(15):1393–9. doi: 10.1212/WNL.0000000000002579 (PMC4831038; doi:10.1212/WNL.0000000000002579)
Supplement: Data Supplement [file supp_86_15_1393__index.html]

Data Supplement 

# Frontotemporal networks and behavioral symptoms in primary progressive aphasia

## Data Supplement

Eight tables and four figures; one PDF file and four .jpeg files.

**Neurology® data supplements are not copyedited before publication. Published editorials and translations have been copyedited.  
 © 2016 American Academy of Neurology.  
  
 Files in this Data Supplement:**

- Tables e-1 to e-8 - PDF file
- Figure e-1 - .jpeg file
- Figure e-2 - .jpeg file
- Figure e-3 - .jpeg file
- Figure e-4 - .jpeg file
