# Supplementary material for: Frontotemporal networks and behavioral symptoms in primary progressive aphasia
Source: Neurology. 2016 Apr 12;86(15):1393–9. doi: 10.1212/WNL.0000000000002579 (PMC4831038; doi:10.1212/WNL.0000000000002579)
Supplement: Data Supplement [file supp_WNL.0000000000002579_Tables.pdf]

Table e-1. Correlations between tract-specific measurements of the tracts of interest and neuropsychiatric assessments in PPA patients.

Tract	DTI	FBI negative scores	FBI positive scores	FBI total scores
**Left UF**	N streamlines	Rho Spearman = -0.460<br>p < 0.001	Rho Spearman = -0.530<br>p = 0.001	Rho Spearman = -0.549<br>p = 0.001
	Fractional anisotropy	Rho Spearman = -0.450<br>p < 0.001	Rho Spearman = -0.500<br>p < 0.001	Rho Spearman = -0.490<br>p < 0.001
	Axial diffusivity	Rho Spearman = 0.575<br>p < 0.001	Rho Spearman = 0.400<br>p < 0.001	Rho Spearman = 0.450<br>p < 0.001
	Perpendicular diffusivity	Rho Spearman = 0.540<br>p < 0.001	Rho Spearman = 0.600<br>p < 0.001	Rho Spearman = 0.540<br>p < 0.001
**Right UF**	N streamlines	Rho Spearman = 0.120<br>p = 0.045	Rho Spearman = -0.038<br>p = 0.915	Rho Spearman = 0.071<br>p = 0.852
	Fractional anisotropy	Rho Spearman = -0.120<br>p = 0.321	Rho Spearman = -0.231<br>p = 0.609	Rho Spearman = -0.321<br>p = 0.432
	Axial diffusivity	Rho Spearman = 0.443<br>p = 0.005	Rho Spearman = 0.432<br>p = 0.005	Rho Spearman = 0.421<br>p = 0.001
	Perpendicular diffusivity	Rho Spearman = 0.497<br>p < 0.001	Rho Spearman = 0.576<br>p < 0.001	Rho Spearman = 0.580<br>p < 0.001
**Left IFOF**	N streamlines	Rho Spearman = 0.111<br>p = 0.787	Rho Spearman = 0.243<br>p = 0.154	Rho Spearman = 0.221<br>p = 0.432
	Fractional anisotropy	Rho Spearman = -0.332<br>p = 0.143	Rho Spearman = -0.076<br>p = 0.667	Rho Spearman = -0.259<br>p = 0.780
	Axial diffusivity	Rho Spearman = 0.456<br>p = 0.235	Rho Spearman = -0.045<br>p = 0.987	Rho Spearman = 0.234<br>p = 0.567
	Perpendicular diffusivity	Rho Spearman = 0.768<br>p = 0.078	Rho Spearman = 0.346<br>p = 0.890	Rho Spearman = 0.789<br>p = 0.567

**Right IFOF**	N streamlines	Rho Spearman = 0.678<br>p = 0.908	Rho Spearman = 0.531<br>p = 0.543	Rho Spearman = 0.721<br>p = 0.772
	Fractional anisotropy	Rho Spearman = -0.523<br>p = 0.852	Rho Spearman = -0.863<br>p = 0.642	Rho Spearman = -0.074<br>p = 0.732
	Axial diffusivity	Rho Spearman = 0.532<br>p = 0.345	Rho Spearman = 0.563<br>p = 0.563	Rho Spearman = 0.341<br>p = 0.145
	Perpendicular diffusivity	Rho Spearman = 0.342<br>p = 0.126	Rho Spearman = 0.231<br>p = 0.453	Rho Spearman = 0.171<br>p = 0.876
**Left ILF**	N streamlines	Rho Spearman = 0.753<br>p = 0.654	Rho Spearman = 0.165<br>p = 0.456	Rho Spearman = 0.176<br>p = 0.765
	Fractional anisotropy	Rho Spearman = -0.564<br>p = 0.900	Rho Spearman = 0.453<br>p = 0.653	Rho Spearman = -0.078<br>p = 0.564
	Axial diffusivity	Rho Spearman = 0.546<br>p = 0.098	Rho Spearman = 0.075<br>p = 0.908	Rho Spearman = 0.234<br>p = 0.567
	Perpendicular diffusivity	Rho Spearman = 0.456<br>p = 0.455	Rho Spearman = -0.567<br>p = 0.564	Rho Spearman = 0.456<br>p = 0.456
**Right ILF**	N streamlines	Rho Spearman = 0.056<br>p = 0.456	Rho Spearman = 0.123<br>p = 0.389	Rho Spearman = 0.245<br>p = 0.489
	Fractional anisotropy	Rho Spearman = -0.345<br>p = 0.134	Rho Spearman = -0.089<br>p = 0.789	Rho Spearman = -0.453<br>p = 0.378
	Axial diffusivity	Rho Spearman = 0.345<br>p = 0.231	Rho Spearman = 0.245<br>p = 0.456	Rho Spearman = 0.345<br>p = 0.567
	Perpendicular diffusivity	Rho Spearman = 0.456<br>p = 0.078	Rho Spearman = 0.678<br>p = 0.890	Rho Spearman = 0.356<br>p = 0.145

UF = Uncinate Fasciculus; IFOF = Inferior Fronto-Occipital Fasciculus; ILF = Inferior Longitudinal Fasciculus; FBI = Frontal Behavioral Inventory.
N streamlines = Number of streamlines

Table e-2. Correlations between cortical thickness analysis of the orbitofrontal and anterior temporal lobe cortices and neuropsychiatric assessments in PPA patients.

**Region**	**FBI negative scores**	**FBI positive scores**	**FBI total scores**
**Left OFC**	Rho Spearman = -0.460<br>p = 0.001	Rho Spearman = -0.523<br>p = 0.001	Rho Spearman = -0.534<br>p = 0.001
**Right OFC**	Rho Spearman = -0.323<br>p = 0.099	Rho Spearman = -0.467<br>p = 0.004	Rho Spearman = -0.379<br>p = 0.015
**Left ATL**	Rho Spearman = -0.521<br>p = 0.001	Rho Spearman = -0.590<br>p < 0.001	Rho Spearman = -0.580<br>p < 0.001
**Right ATL**	Rho Spearman = -0.332<br>p = 0.049	Rho Spearman = -0.499<br>p = 0.004	Rho Spearman = -0.467<br>p = 0.010

FBI = Frontal Behavioral Inventory; OFC = orbitofrontal cortex; ATL= anterior temporal lobe cortex.

Table e-3. Correlations between cortical thickness analysis of the left orbitofrontal and left anterior temporal lobe cortices and tract-specific measurements of the tracts of interest in the left hemisphere.

		Left OFC	Left ATL
**Left Uncinate fasciculus**	N streamlines	Rho Spearman = 0.324<br>p = 0.125	Rho Spearman = 0.567<br>p = 0.004
	Fractional anisotropy	Rho Spearman = 0.524<br>p = 0.001	Rho Spearman = 0.488<br>p = 0.003
	Axial diffusivity	Rho Spearman = -0.378<br>p = 0.007	Rho Spearman = -0.570<br>p = 0.001
	Perpendicular diffusivity	Rho Spearman = -0.513<br>p = 0.002	Rho Spearman = -0.580<br>p = 0.001
**Left Inferior frontooccipital fasciculus**	N streamlines	Rho Spearman = 0.008<br>p = 0.689	Rho Spearman = 0.089<br>p = 0.956
	Fractional anisotropy	Rho Spearman = 0.342<br>p = 0.132	Rho Spearman = 0.035<br>p = 0.918
	Axial diffusivity	Rho Spearman = -0.090<br>p = 0.943	Rho Spearman = -0.090<br>p = 0.678
	Perpendicular diffusivity	Rho Spearman = -0.453<br>p = 0.178	Rho Spearman = 0.089<br>p = 0.900
**Left Inferior longitudinal fasciculus**	N streamlines	Rho Spearman = -0.345<br>p = 0.234	Rho Spearman = -0.278<br>p = 0.342
	Fractional anisotropy	Rho Spearman = -0.198<br>p = 0.456	Rho Spearman = -0.147<br>p = 0.564
	Axial diffusivity	Rho Spearman = -0.089<br>p = 0.456	Rho Spearman = -0.189<br>p = 0.390
	Perpendicular diffusivity	Rho Spearman = 0.089<br>p = 0.889	Rho Spearman = -0.078<br>p = 0.990

OFC = orbitofrontal cortex; ATL= anterior temporal lobe cortex.
N streamlines = Number of streamlines

Table e-4. Correlations between cortical thickness analysis of the right orbitofrontal and right anterior temporal lobe cortices and tract-specific measurements of the tracts of interest in the right hemisphere.

		**Right OFC**	**Right ATL**
**Right Uncinate fasciculus**	N streamlines	Rho Spearman = 0.023<br>p = 0.901	Rho Spearman = 0.236<br>p = 0.187
	Fractional anisotropy	Rho Spearman = 0.043<br>p = 0.812	Rho Spearman = 0.067<br>p = 0.712
	Axial diffusivity	Rho Spearman = -0.007<br>p = 0.968	Rho Spearman = -0.464<br>p = 0.007
	Perpendicular diffusivity	Rho Spearman = -0.207<br>p = 0.248	Rho Spearman = -0.615<br>p < 0.001
**Right Inferior frontooccipital fasciculus**	N streamlines	Rho Spearman = -0.036<br>p = 0.841	Rho Spearman = -0.181<br>p = 0.312
	Fractional anisotropy	Rho Spearman = -0.140<br>p = 0.438	Rho Spearman = 0.085<br>p = 0.639
	Axial diffusivity	Rho Spearman = 0.023<br>p = 0.899	Rho Spearman = -0.011<br>p = 0.953
	Perpendicular diffusivity	Rho Spearman = 0.077<br>p = 0.668	Rho Spearman = -0.106<br>p = 0.557
**Right Inferior longitudinal fasciculus**	N streamlines	Rho Spearman = -0.347<br>p = 0.048	Rho Spearman = -0.157<br>p = 0.384
	Fractional anisotropy	Rho Spearman = -0.076<br>p = 0.672	Rho Spearman = 0.118<br>p = 0.514
	Axial diffusivity	Rho Spearman = 0.309<br>p = 0.081	Rho Spearman = -0.148<br>p = 0.412
	Perpendicular diffusivity	Rho Spearman = 0.211<br>p = 0.238	Rho Spearman = -0.189<br>p = 0.292

OFC = orbitofrontal cortex; ATL= anterior temporal lobe cortex.
N streamlines = Number of streamlines

Table e-5. Correlations between tract-specific measurements of the tracts of interest and neuropsychiatric assessments in patients with Logopenic variant of Primary Progressive Aphasia.

Tract	DTI	FBI negative scores	FBI positive scores	FBI total scores
**Left UF**	N streamlines	Rho Spearman = -0.636<br>p = 0.124	Rho Spearman = -0.128<br>p = 0.784	Rho Spearman = -0.667<br>p = 0.102
	Fractional anisotropy	Rho Spearman = -0.527<br>p = 0.224	Rho Spearman = -0.385<br>p = 0.393	Rho Spearman = -0.414<br>p = 0.355
	Axial diffusivity	Rho Spearman = 0.927<br>p = 0.003	Rho Spearman = 0.055<br>p = 0.907	Rho Spearman = 0.847<br>p = 0.016
	Perpendicular diffusivity	Rho Spearman = 0.782<br>p = 0.038	Rho Spearman = -0.092<br>p = 0.845	Rho Spearman = 0.775<br>p = 0.041
**Right UF**	N streamlines	Rho Spearman = -0.459<br>p = 0.301	Rho Spearman = -0.426<br>p = 0.341	Rho Spearman = -0.691<br>p = 0.086
	Fractional anisotropy	Rho Spearman = -0.655<br>p = 0.111	Rho Spearman = 0.165<br>p = 0.723	Rho Spearman = -0.631<br>p = 0.129
	Axial diffusivity	Rho Spearman = 0.872<br>p = 0.011	Rho Spearman = -0.019<br>p = 0.969	Rho Spearman = -0.631<br>p = 0.129
	Perpendicular diffusivity	Rho Spearman = 0.746<br>p = 0.054	Rho Spearman = -0.165<br>p = 0.723	Rho Spearman = 0.685<br>p = 0.090
**Left IFOF**	N streamlines	Rho Spearman = -0.036<br>p = 0.938	Rho Spearman = 0.147<br>p = 0.753	Rho Spearman = -0.108<br>p = 0.818
	Fractional anisotropy	Rho Spearman = -0.600<br>p = 0.154	Rho Spearman = 0.422<br>p = 0.345	Rho Spearman = -0.505<br>p = 0.248
	Axial diffusivity	Rho Spearman = 0.709<br>p = 0.074	Rho Spearman = 0.000<br>p = 1.000	Rho Spearman = 0.450<br>p = 0.310
	Perpendicular diffusivity	Rho Spearman = 0.873<br>p = 0.010	Rho Spearman = -0.073<br>p = 0.876	Rho Spearman = 0.811<br>p = 0.027
**Right IFOF**	N streamlines	Rho Spearman = 0.073<br>p = 0.877	Rho Spearman = -0.239<br>p = 0.606	Rho Spearman = -0.018<br>p = 0.969
	Fractional anisotropy	Rho Spearman = -0.800<br>p = 0.031	Rho Spearman = 0.220<br>p = 0.635	Rho Spearman = -0.739<br>p = 0.058
	Axial diffusivity	Rho Spearman = 0.455<br>p = 0.305	Rho Spearman = 0.128<br>p = 0.784	Rho Spearman = 0.252<br>p = 0.585
	Perpendicular diffusivity	Rho Spearman = 0.818<br>p = 0.024	Rho Spearman = -0.018<br>p = 0.969	Rho Spearman = 0.757<br>p = 0.049
**Left ILF**	N streamlines	Rho Spearman = -0.436<br>p = 0.328	Rho Spearman = -0.771<br>p = 0.042	Rho Spearman = -0.631<br>p = 0.129
	Fractional anisotropy	Rho Spearman = -0.873<br>p = 0.100	Rho Spearman = 0.220<br>p = 0.635	Rho Spearman = -0.739<br>p = 0.058
	Axial diffusivity	Rho Spearman = 0.491<br>p = 0.263	Rho Spearman = 0.018<br>p = 0.969	Rho Spearman = 0.559<br>p = 0.192

	Perpendicular diffusivity	Rho Spearman = 0.800<br>p = 0.310	Rho Spearman = -0.147<br>p = 0.753	Rho Spearman = 0.703<br>p = 0.078
**Right ILF**	N streamlines	Rho Spearman = -0.109<br>p = 0.816	Rho Spearman = 0.385<br>p = 0.383	Rho Spearman = 0.180<br>p = 0.699
	Fractional anisotropy	Rho Spearman = -0.691<br>p = 0.086	Rho Spearman = 0.312<br>p = 0.496	Rho Spearman = -0.505<br>p = 0.248
	Axial diffusivity	Rho Spearman = 0.509<br>p = 0.243	Rho Spearman = 0.330<br>p = 0.469	Rho Spearman = 0.414<br>p = 0.355
	Perpendicular diffusivity	Rho Spearman = 0.891<br>p = 0.070	Rho Spearman = -0.018<br>p = 0.969	Rho Spearman = 0.757<br>p = 0.049

UF = Uncinate Fasciculus; IFOF = Inferior Fronto-Occipital Fasciculus; ILF = Inferior Longitudinal Fasciculus; FBI = Frontal Behavioral Inventory.
N streamlines = Number of streamlines

Table e-6. Correlations between tract-specific measurements of the tracts of interest and neuropsychiatric assessments in patients with Agrammatic variant of Primary Progressive Aphasia.

Tract	DTI	FBI negative scores	FBI positive scores	FBI total scores
**Left UF**	N streamlines	Rho Spearman = -0.429 p = 0.397	Rho Spearman = -0.309 p = 0.552	Rho Spearman = -0.429 p = 0.397
	Fractional anisotropy	Rho Spearman = -0.543 p = 0.266	Rho Spearman = -0.309 p = 0.552	Rho Spearman = -0.543 p = 0.266
	Axial diffusivity	Rho Spearman = -0.486 p = 0.329	Rho Spearman = -0.494 p = 0.320	Rho Spearman = -0.486 p = 0.329
	Perpendicular diffusivity	Rho Spearman = 0.371 p = 0.468	Rho Spearman = 0.154 p = 0.770	Rho Spearman = 0.371 p = 0.468
**Right UF**	N streamlines	Rho Spearman = 0.029 p = 0.957	Rho Spearman = 0.093 p = 0.862	Rho Spearman = 0.029 p = 0.957
	Fractional anisotropy	Rho Spearman = -0.600 p = 0.208	Rho Spearman = -0.617 p = 0.192	Rho Spearman = -0.600 p = 0.208
	Axial diffusivity	Rho Spearman = -0.429 p = 0.397	Rho Spearman = -0.247 p = 0.637	Rho Spearman = -0.429 p = 0.397
	Perpendicular diffusivity	Rho Spearman = 0.200 p = 0.704	Rho Spearman = 0.154 p = 0.770	Rho Spearman = 0.200 p = 0.704
**Left IFOF**	N streamlines	Rho Spearman = 0.771 p = 0.072	Rho Spearman = 0.617 p = 0.192	Rho Spearman = 0.771 p = 0.072
	Fractional anisotropy	Rho Spearman = -0.143 p = 0.787	Rho Spearman = -0.031 p = 0.954	Rho Spearman = -0.143 p = 0.787
	Axial diffusivity	Rho Spearman = 0.086 p = 0.872	Rho Spearman = 0.309 p = 0.552	Rho Spearman = 0.086 p = 0.872
	Perpendicular diffusivity	Rho Spearman = 0.145 p = 0.784	Rho Spearman = 0.250 p = 0.632	Rho Spearman = 0.145 p = 0.784
**Right IFOF**	N streamlines	Rho Spearman = 0.371 p = 0.468	Rho Spearman = 0.093 p = 0.862	Rho Spearman = 0.371 p = 0.468
	Fractional anisotropy	Rho Spearman = -0.829 p = 0.420	Rho Spearman = -0.617 p = 0.192	Rho Spearman = -0.829 p = 0.042
	Axial diffusivity	Rho Spearman = 0.029 p = 0.957	Rho Spearman = 0.154 p = 0.770	Rho Spearman = 0.029 p = 0.957
	Perpendicular diffusivity	Rho Spearman = 0.600 p = 0.208	Rho Spearman = 0.463 p = 0.355	Rho Spearman = 0.600 p = 0.208
**Left ILF**	N streamlines	Rho Spearman = 0.314 p = 0.544	Rho Spearman = 0.494 p = 0.320	Rho Spearman = 0.371 p = 0.468
	Fractional anisotropy	Rho Spearman = -0.371 p = 0.468	Rho Spearman = -0.062 p = 0.908	Rho Spearman = -0.371 p = 0.468
	Axial diffusivity	Rho Spearman = -0.200 p = 0.704	Rho Spearman = 0.062 p = 0.908	Rho Spearman = -0.200 p = 0.704
	Perpendicular diffusivity	Rho Spearman = -0.086 p = 0.872	Rho Spearman = -0.247 p = 0.637	Rho Spearman = -0.086 p = 0.872

**Right ILF**	N streamlines	Rho Spearman = 0.116<br>p = 0.827	Rho Spearman = 0.000<br>p = 1.000	Rho Spearman = 0.116<br>p = 0.827
	Fractional anisotropy	Rho Spearman = -0.771<br>p = 0.072	Rho Spearman = -0.617<br>p = 0.192	Rho Spearman = -0.771<br>p = 0.072
	Axial diffusivity	Rho Spearman = 0.429<br>p = 0.397	Rho Spearman = 0.617<br>p = 0.192	Rho Spearman = 0.429<br>p = 0.397
	Perpendicular diffusivity	Rho Spearman = 0.314<br>p = 0.544	Rho Spearman = 0.309<br>p = 0.552	Rho Spearman = 0.314<br>p = 0.544

UF = Uncinate Fasciculus; IFOF = Inferior Fronto-Occipital Fasciculus; ILF = Inferior Longitudinal Fasciculus; FBI = Frontal Behavioral Inventory.
N streamlines = Number of streamlines

Table e-7. Correlations between tract-specific measurements of the tracts of interest and neuropsychiatric assessments in patients with Semantic variant of Primary Progressive Aphasia.

Tract	DTI	FBI negative scores	FBI positive scores	FBI total scores
**Left UF**	N streamlines	Rho Spearman = -0.429<br>p = 0.397	Rho Spearman = -0.257<br>p = 0.623	Rho Spearman = -0.257<br>p = 0.623
	Fractional anisotropy	Rho Spearman = -0.486<br>p = 0.329	Rho Spearman = -0.371<br>p = 0.468	Rho Spearman = -0.371<br>p = 0.468
	Axial diffusivity	Rho Spearman = 0.714<br>p = 0.111	Rho Spearman = 0.771<br>p = 0.072	Rho Spearman = 0.771<br>p = 0.072
	Perpendicular diffusivity	Rho Spearman = 0.543<br>p = 0.266	Rho Spearman = 0.600<br>p = 0.208	Rho Spearman = 0.543<br>p = 0.266
**Right UF**	N streamlines	Rho Spearman = -0.314<br>p = 0.544	Rho Spearman = -0.429<br>p = 0.397	Rho Spearman = -0.314<br>p = 0.544
	Fractional anisotropy	Rho Spearman = -0.429<br>p = 0.397	Rho Spearman = -0.257<br>p = 0.623	Rho Spearman = -0.257<br>p = 0.623
	Axial diffusivity	Rho Spearman = 0.829<br>p = 0.042	Rho Spearman = 0.829<br>p = 0.042	Rho Spearman = 0.886<br>p = 0.019
	Perpendicular diffusivity	Rho Spearman = 0.429<br>p = 0.397	Rho Spearman = 0.257<br>p = 0.623	Rho Spearman = 0.257<br>p = 0.623
**Left IFOF**	N streamlines	Rho Spearman = 0.600<br>p = 0.208	Rho Spearman = -0.200<br>p = 0.704	Rho Spearman = 0.257<br>p = 0.623
	Fractional anisotropy	Rho Spearman = 0.543<br>p = 0.266	Rho Spearman = 0.486<br>p = 0.329	Rho Spearman = 0.657<br>p = 0.156
	Axial diffusivity	Rho Spearman = 0.677<br>p = 0.344	Rho Spearman = 0.600<br>p = 0.208	Rho Spearman = 0.886<br>p = 0.019
	Perpendicular diffusivity	Rho Spearman = 0.257<br>p = 0.623	Rho Spearman = 0.029<br>p = 0.957	Rho Spearman = 0.029<br>p = 0.957
**Right IFOF**	N streamlines	Rho Spearman = -0.029<br>p = 0.957	Rho Spearman = 0.086<br>p = 0.872	Rho Spearman = 0.143<br>p = 0.787
	Fractional anisotropy	Rho Spearman = 0.200<br>p = 0.704	Rho Spearman = 0.029<br>p = 0.957	Rho Spearman = 0.257<br>p = 0.623
	Axial diffusivity	Rho Spearman = 0.829<br>p = 0.042	Rho Spearman = 0.257<br>p = 0.623	Rho Spearman = 0.657<br>p = 0.156
	Perpendicular diffusivity	Rho Spearman = 0.600<br>p = 0.208	Rho Spearman = 0.029<br>p = 0.957	Rho Spearman = 0.257<br>p = 0.623
**Left ILF**	N streamlines	Rho Spearman = 0.657<br>p = 0.156	Rho Spearman = 0.200<br>p = 0.704	Rho Spearman = 0.543<br>p = 0.266
	Fractional anisotropy	Rho Spearman = 0.257<br>p = 0.623	Rho Spearman = 0.029<br>p = 0.957	Rho Spearman = 0.200<br>p = 0.704
	Axial diffusivity	Rho Spearman = 0.543<br>p = 0.266	Rho Spearman = 0.714<br>p = 0.111	Rho Spearman = 0.771<br>p = 0.072
	Perpendicular diffusivity	Rho Spearman = -0.200<br>p = 0.704	Rho Spearman = 0.200<br>p = 0.704	Rho Spearman = -0.029<br>p = 0.957
**Right ILF**	N streamlines	Rho Spearman = -0.314<br>p = 0.544	Rho Spearman = -0.086<br>p = 0.872	Rho Spearman = -0.143<br>p = 0.787
	Fractional anisotropy	Rho Spearman = -0.371	Rho Spearman = -0.086<br>p = 0.872	Rho Spearman = -0.143<br>p = 0.787

		p = 0.468		
	Axial diffusivity	Rho Spearman = 0.886<br>p = 0.019	Rho Spearman = 0.657<br>p = 0.156	Rho Spearman = 0.886<br>p = 0.019
	Perpendicular diffusivity	Rho Spearman = 0.429<br>p = 0.397	Rho Spearman = 0.314<br>p = 0.544	Rho Spearman = 0.314<br>p = 0.544

UF = Uncinate Fasciculus; IFOF = Inferior Fronto-Occipital Fasciculus; ILF = Inferior Longitudinal Fasciculus; FBI = Frontal Behavioral Inventory.
N streamlines = Number of streamlines

Table e-8. Correlations between tract-specific measurements of the tracts of interest and neuropsychiatric assessments in patients with unclassified variant of Primary Progressive Aphasia.

Tract	DTI	FBI negative scores	FBI positive scores	FBI total scores
**Left UF**	N streamlines	Rho Spearman = -0.198 p = 0.670	Rho Spearman = -0.158 p = 0.736	Rho Spearman = -0.162 p = 0.728
	Fractional anisotropy	Rho Spearman = -0.198 p = 0.670	Rho Spearman = 0.729 p = 0.063	Rho Spearman = -0.054 p = 0.908
	Axial diffusivity	Rho Spearman = 0.468 p = 0.289	Rho Spearman = 0.020 p = 0.967	Rho Spearman = 0.505 p = 0.248
	Perpendicular diffusivity	Rho Spearman = 0.414 p = 0.355	Rho Spearman = -0.315 p = 0.491	Rho Spearman = 0.378 p = 0.403
**Right UF**	N streamlines	Rho Spearman = -0.775 p = 0.041	Rho Spearman = -0.512 p = 0.240	Rho Spearman = -0.847 p = 0.016
	Fractional anisotropy	Rho Spearman = -0.108 p = 0.818	Rho Spearman = 0.059 p = 0.900	Rho Spearman = -0.144 p = 0.758
	Axial diffusivity	Rho Spearman = 0.721 p = 0.068	Rho Spearman = 0.118 p = 0.801	Rho Spearman = 0.757 p = 0.049
	Perpendicular diffusivity	Rho Spearman = 0.414 p = 0.355	Rho Spearman = 0.118 p = 0.801	Rho Spearman = 0.487 p = 0.268
**Left IFOF**	N streamlines	Rho Spearman = -0.757 p = 0.490	Rho Spearman = -0.217 p = 0.641	Rho Spearman = -0.793 p = 0.333
	Fractional anisotropy	Rho Spearman = -0.378 p = 0.403	Rho Spearman = 0.591 p = 0.162	Rho Spearman = -0.270 p = 0.558
	Axial diffusivity	Rho Spearman = 0.468 p = 0.289	Rho Spearman = -0.158 p = 0.736	Rho Spearman = 0.432 p = 0.333
	Perpendicular diffusivity	Rho Spearman = 0.216 p = 0.641	Rho Spearman = -0.493 p = 0.261	Rho Spearman = 0.144 p = 0.758
**Right IFOF**	N streamlines	Rho Spearman = -0.378 p = 0.403	Rho Spearman = 0.256 p = 0.579	Rho Spearman = -0.342 p = 0.452
	Fractional anisotropy	Rho Spearman = -0.324 p = 0.478	Rho Spearman = 0.256 p = 0.579	Rho Spearman = -0.288 p = 0.531
	Axial diffusivity	Rho Spearman = 0.396 p = 0.379	Rho Spearman = 0.374 p = 0.408	Rho Spearman = 0.468 p = 0.289
	Perpendicular diffusivity	Rho Spearman = 0.432 p = 0.333	Rho Spearman = 0.079 p = 0.867	Rho Spearman = 0.468 p = 0.289
**Left ILF**	N streamlines	Rho Spearman = 0.144 p = 0.758	Rho Spearman = -0.729 p = 0.063	Rho Spearman = 0.000 p = 1.000
	Fractional anisotropy	Rho Spearman = -0.162 p = 0.728	Rho Spearman = 0.394 p = 0.382	Rho Spearman = -0.126 p = 0.788
	Axial diffusivity	Rho Spearman = 0.360 p = 0.427	Rho Spearman = -0.335 p = 0.463	Rho Spearman = 0.252 p = 0.585
	Perpendicular diffusivity	Rho Spearman = 0.108 p = 0.818	Rho Spearman = -0.394 p = 0.382	Rho Spearman = 0.072 p = 0.878
**Right ILF**	N streamlines	Rho Spearman = -0.109 p = 0.816	Rho Spearman = -0.716 p = 0.071	Rho Spearman = -0.109 p = 0.816

	Fractional anisotropy	Rho Spearman = -0.378<br>p = 0.403	Rho Spearman = 0.591<br>p = 0.162	Rho Spearman = -0.270<br>p = 0.558
	Axial diffusivity	Rho Spearman = 0.234<br>p = 0.613	Rho Spearman = 0.236<br>p = 0.610	Rho Spearman = 0.306<br>p = 0.504
	Perpendicular diffusivity	Rho Spearman = 0.162<br>p = 0.728	Rho Spearman = -0.059<br>p = 0.900	Rho Spearman = 0.198<br>p = 0.670

UF = Uncinate Fasciculus; IFOF = Inferior Fronto-Occipital Fasciculus; ILF = Inferior Longitudinal Fasciculus; FBI = Frontal Behavioral Inventory.
N streamlines = Number of streamlines
