# Supplementary figures and images for: Frontotemporal networks and behavioral symptoms in primary progressive aphasia
Source: Neurology. 2016 Apr 12;86(15):1393–9. doi: 10.1212/WNL.0000000000002579 (PMC4831038; doi:10.1212/WNL.0000000000002579)

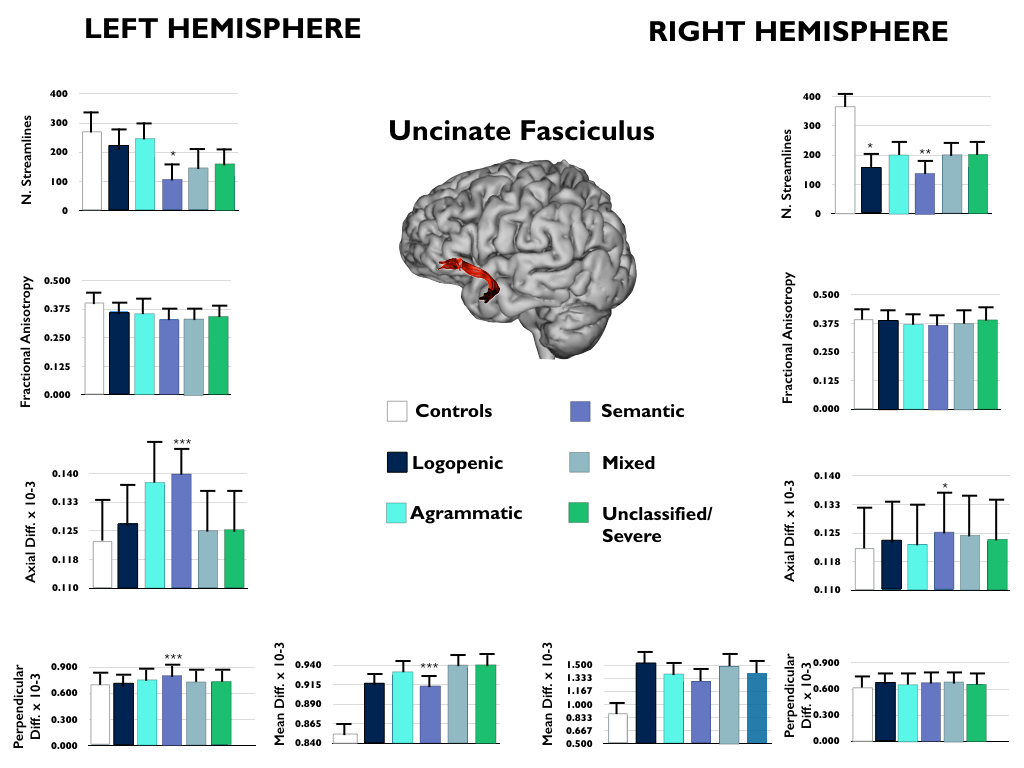

Supplement: Data Supplement [file supp_WNL.0000000000002579_Figure_e-1.jpg]

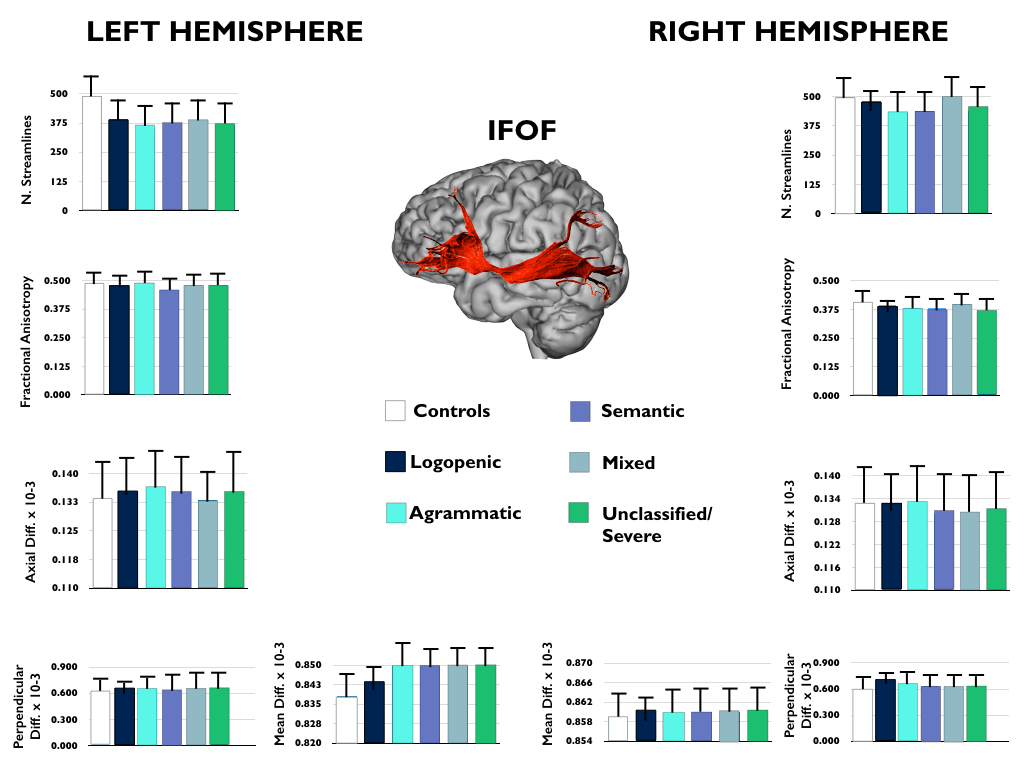

Supplement: Data Supplement [file supp_WNL.0000000000002579_Figure_e-2.jpg]

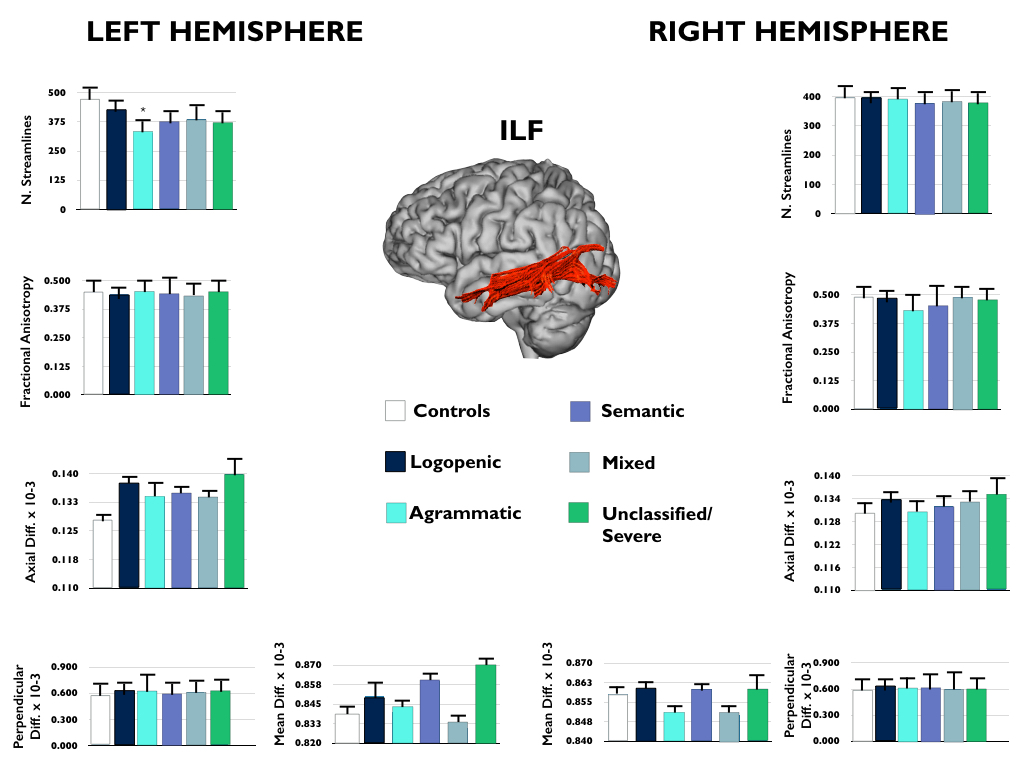

Supplement: Data Supplement [file supp_WNL.0000000000002579_Figure_e-3.jpg]

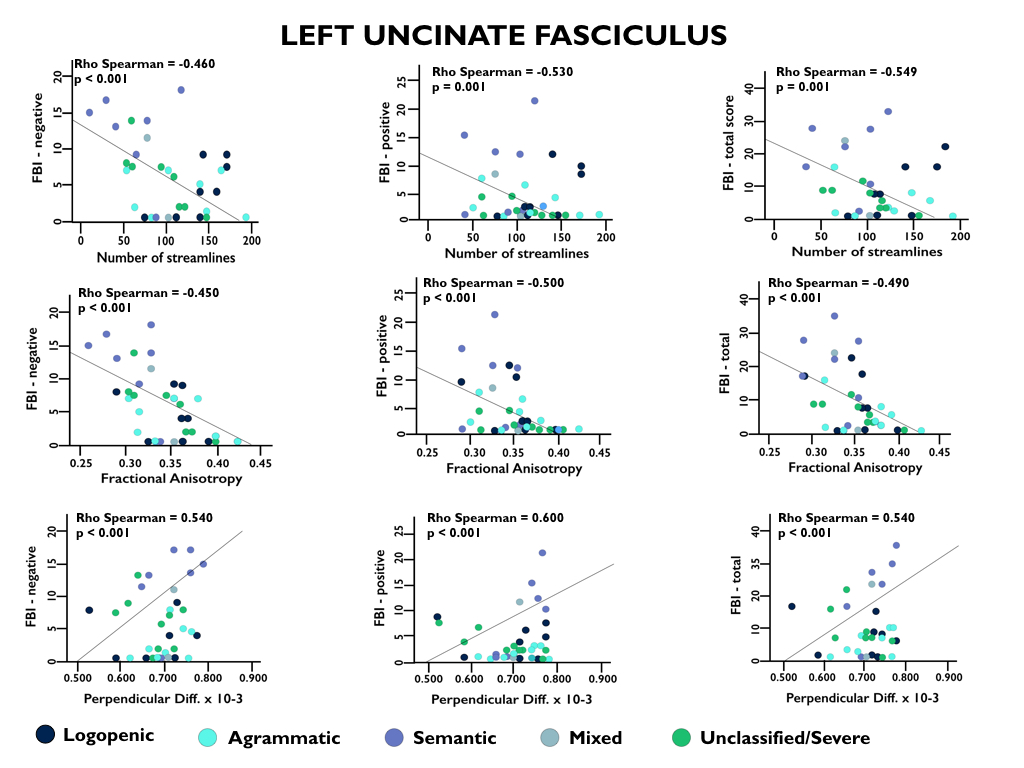

Supplement: Data Supplement [file supp_WNL.0000000000002579_Figure_e-4.jpg]
